# Intravital single-molecule imaging reveals cytoskeletal turnover as a driver of membrane remodeling in live animals

**DOI:** 10.64898/2026.02.25.708035

**Published:** 2026-02-25

**Authors:** Marco Heydecker, Desu Chen, Andrius Masedunskas, Melissa Mikolaj, Kedar Narayan, Jiji Chen, Harshad Vishwasrao, Tobias Meckel, Edna Hardeman, Peter Gunning, Roberto Weigert

**Affiliations:** 1 Laboratory of Cellular and Molecular Biology, Center for Cancer Research, National Cancer Institute, National Institutes of Health, Bethesda, MD, USA; 2 School of Biomedical Sciences, University of New South Wales Sydney, Sydney, NSW, Australia; 3 Center for Molecular Microscopy, Center for Cancer Research, National Cancer Institute, National Institutes of Health, Bethesda, MD, USA and Cancer Research Technology Program, Frederick National Laboratory for Cancer Research, Frederick, MD, USA.; 4 Advanced Imaging and Microscopy Resource, National Institute of Biomedical Imaging and Bioengineering, National Institutes of Health, Bethesda, MD, USA; 5 Department of Chemistry, Macromolecular Chemistry & Paper Chemistry, Technical University Darmstadt, Alarich-Weiss-Straße 8, 64287, Darmstadt, Germany

## Abstract

Understanding how cells regulate plasma membrane architecture inside intact living organs in a live animal has been limited by the inability to directly measure molecular dynamics in vivo. Here we introduce intravital single-molecule microscopy (iSiMM), an imaging approach that enables tracking of individual, endogenously expressed cytoskeletal components at the plasma membrane in live mice. Applying iSiMM to murine acinar secretory cells, we identify discrete basolateral membrane domains built on deeply folded membrane infolds that function as a pre-existing membrane reservoir. Single-molecule measurements reveal continuous, regulated molecular turnover within these domains. Physiological stimulation accelerates cytoskeletal exchange promoting rapid membrane unfolding and cell expansion. Together, these findings establish iSiMM as a general strategy for probing molecular kinetics underlying dynamic cellular behaviors in intact organs.

## One-sentence summary

Intravital single-molecule microscopy enables direct measurement of molecular kinetics underlying dynamic cellular behaviors in intact living organs.

## Introduction

The plasma membrane is a mechanically active interface whose organization and dynamics are central to epithelial physiology, secretion, and volume regulation (Diz-Muñoz et al., 2018; Renne and Ernst, 2023; Wang et al., 2023). These functions are governed by the actomyosin cortex, whose assembly, turnover, and force-generating properties determine membrane tension, viscoelastic behavior, and mechanical responsiveness (De Belly et al., 2023; Lieber et al., 2013). While these processes have been extensively characterized in cultured cells and reconstituted systems, their kinetic behavior inside living mammals remains largely unexplored. In particular, there is little direct information on how cytoskeletal proteins bind, release, and reorganize at the plasma membrane within intact organs of live animals, where tissue architecture, mechanical constraints, and physiological regulation differ fundamentally from cell culture models (Brähler et al., 2016; Ebrahim et al., 2019).

Salivary gland acinar cells provide a compelling physiological context in which this gap becomes evident. During β-adrenergic stimulation, acinar cells undergo rapid and reversible volume expansion of approximately 15%, requiring a substantial increase in basolateral membrane surface area (Catalán et al., 2015). This expansion could, in principle, be achieved through basolateral exocytosis or recruitment of a pre-existing membrane reservoir (Figard and Sokac, 2014). However, secretory granules fuse exclusively at the apical membrane and are rapidly retrieved by compensatory endocytosis (Masedunskas et al., 2011b; Weigert et al., 2010), and prior intravital imaging studies have not detected basolateral exocytic events during stimulation (Ebrahim et al., 2019; Heydecker et al., 2024; Masedunskas et al., 2011a). Together, these observations suggest that acinar cells rely on a mechanism of membrane deployment that does not involve bulk membrane addition, yet the structural basis and molecular regulation of such a mechanism have remained unresolved.

Addressing this problem requires the ability to directly observe molecular dynamics at the plasma membrane inside a living mammal. To overcome this limitation, we developed intravital single-molecule microscopy (iSiMM), an imaging strategy that enables direct measurement of molecular binding, diffusion, and turnover of endogenously expressed cytoskeletal proteins inside live mice. By combining intravital subcellular microscopy (ISMic, (Ebrahim et al., 2019; Heydecker et al., 2024; Masedunskas et al., 2018), shallow-angle illumination (Tokunaga et al., 2008), and single-molecule tracking, iSiMM allows quantitative interrogation of molecular kinetics at the basolateral membrane in intact organs in vivo. Here, we apply iSiMM to salivary gland acinar cells in live mice to uncover the molecular principles governing basolateral membrane deployment during physiological stimulation. Using ISMic and ultrastructural analysis, we identify discrete basolateral membrane domains built on deeply folded membrane infolds that function as a membrane reservoir. iSiMM reveals that nonmuscle myosin II isoforms and Tropomyosin 3.1 undergo rapid, regulated binding–unbinding cycles within these domains, and that β-adrenergic stimulation accelerates myosin turnover to promote membrane unfolding. Together, these findings establish iSiMM as a general strategy for linking molecular kinetics to membrane mechanics inside living animals and reveal a kinetic mechanism by which epithelial cells dynamically regulate plasma membrane surface area under physiological conditions.

## Results

### Intravital single-molecule imaging reveals dynamic NMII organization at the basolateral membrane

**Figure 1.**
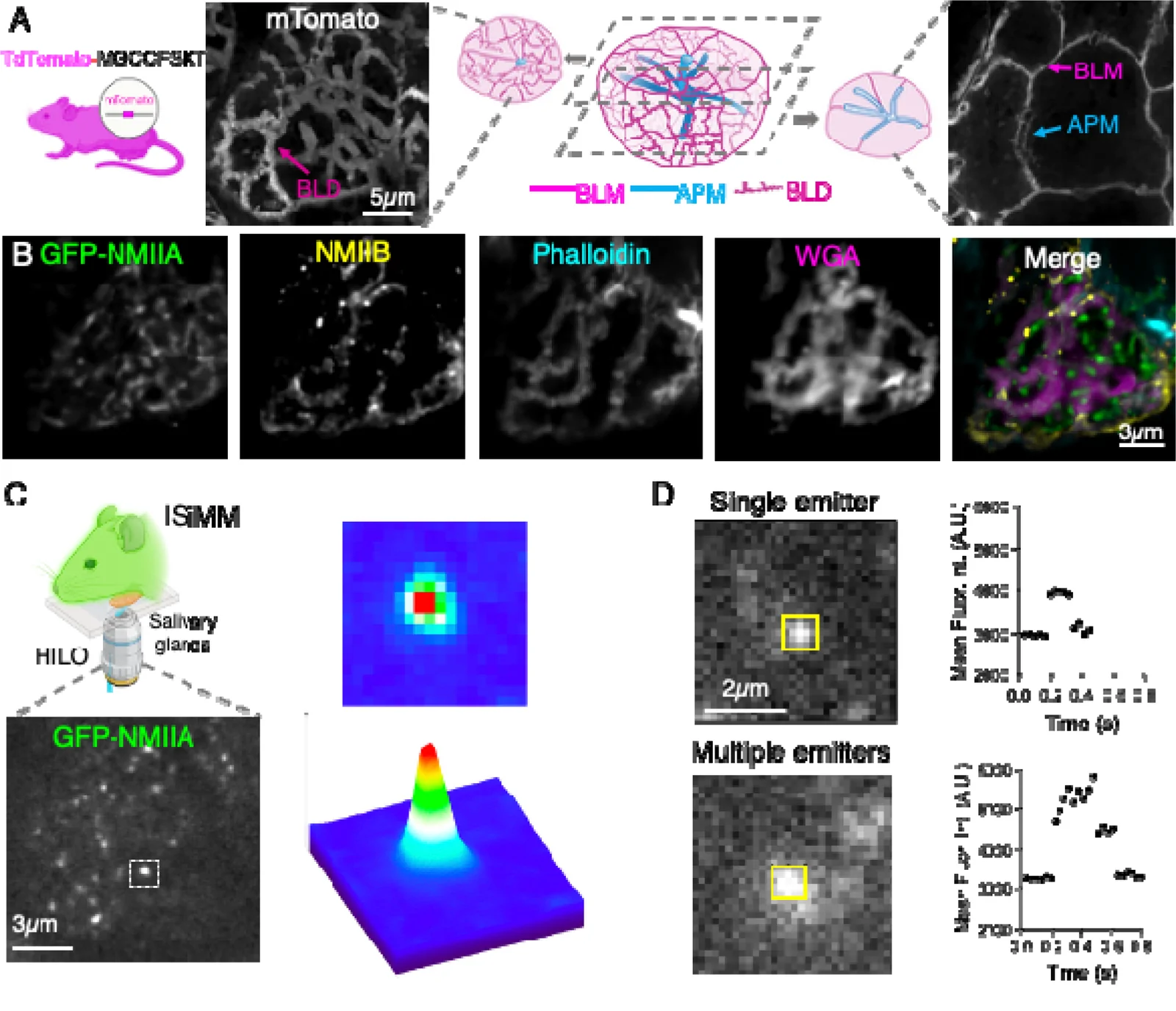
Basolateral membrane domains are associated with the actomyosin cytoskeleton and are accessible to intravital single-molecule imaging. (A) Left, schematic illustrating the mTom mouse model and the structure of the membrane-targeted tdTomato reporter. Right, representative single optical sections from a z-stack acquired by spinning-disk intravital microscopy after anesthesia and surgical exposure of the salivary glands in mTom mice (see Material and Methods; Movie S1). The left image shows BLDs visible at the tissue surface, while the right image, acquired at the same depth, shows both the basolateral and the apical plasma membrane within the acinar plane. (B) Immunofluorescence analysis of salivary glands from GFP–NMIIA mice. Animals were euthanized, glands excised, and tissue processed for immunofluorescence to label GFP-NMIIA (green), NMIIB (yellow), F-actin (phalloidin, cyan), and wheat germ agglutinin (WGA, magenta) as described in the Material and Methods. Actin and NMII filaments are enriched within BLDs. Scale bar, 3 μm. (C) Schematic of the intravital imaging configuration using highly inclined and laminated optical sheet (HILO) illumination to enable intravital single-molecule microscopy (iSiMM) of BLDs in GFP-NMIIA mice. Individual fluorescent spots exhibit a Gaussian intensity distribution (inset). Scale bar, 3 μm. (D) Representative fluorescence intensity traces of GFP-NMIIA over time (yellow boxed regions), illustrating both single- and multiple-emitter events. Stepwise changes in intensity allow discrimination between single and multiple fluorescent emitters during iSiMM acquisition.

To assess the organization of the basolateral membrane (BLM) in vivo, we first imaged salivary glands from mice expressing the membrane-targeted reporter mTomato (mTom) ((Muzumdar et al., 2007), Fig. 1A). Intravital subcellular microscopy (ISMic) revealed that the BLM is not homogeneous but instead organized into discrete basolateral membrane domains (BLDs) interspersed with areas of lower signal intensity (Fig. 1A, Movie S1). These BLDs were consistently observed across acini and persisted over tens of minutes, maintaining their relative position and shape during time-lapse imaging, indicating a stable underlying organization (Movie S2).

To confirm that BLDs represent bona fide basolateral membrane specializations and are not an artifact of the reporter, we used complementary labeling strategies. In mTom-expressing mice, wheat germ agglutinin (WGA, (Chang et al., 1975)) labeling overlapped with mTom-positive BLDs (Fig. 1B, right panel; Fig. S1A, left panel). In wild-type mice lacking any membrane reporter, WGA labeling colocalized with Na^+^/K^+^-ATPase, a canonical basolateral membrane marker (Rodriguez-Boulan and Macara, 2014), confirming that these structures correspond to authentic BLM domains and are independent of the membrane reporter used (Fig. S1A, right panel). We next examined the cytoskeletal composition of BLDs. Fixed glands were stained with phalloidin together with antibodies against NMIIA and NMIIB (Fig. 1B). F-actin, NMIIA, and NMIIB were all enriched within BLDs, demonstrating that these domains are associated with an actomyosin network. Quantitative colocalization analysis revealed a high degree of alignment between membrane signal and F-actin (Pearson’s coefficient 0.59 ± 0.09), with lower but significant correlations for NMIIA (0.29 ± 0.09) and NMIIB (0.32 ± 0.11) (Fig. S1B).

Line-scan analyses further showed that NMIIA and NMIIB localize adjacent to actin filament bundles rather than directly overlapping with peak actin signal, consistent with a peripheral association with cortical actin structures (Fig. S2B). Although BLDs appear morphologically stable at the micron scale, their molecular dynamics cannot be inferred from ensemble imaging. To probe actomyosin behavior at the single-molecule level, we implemented intravital single-molecule microscopy (iSiMM) using highly inclined and laminated optical sheet (HILO) illumination (Tokunaga et al., 2008). In salivary glands, the BLM is the first cellular structure encountered from the tissue surface, lying at a depth of approximately 15–30 μm, making it accessible to shallow-angle excitation. Using GFP-NMIIAknock-in mice (Wang et al., 2010; Zhang et al., 2012), individual GFP–NMIIA molecules could be selectively visualized at the basal surface of surgically exteriorized glands (Fig. 1C; Movie S3). Fluorescence intensity traces exhibited stepwise photobleaching behavior consistent with single emitters (Fig. 1D).

**Figure 2.**
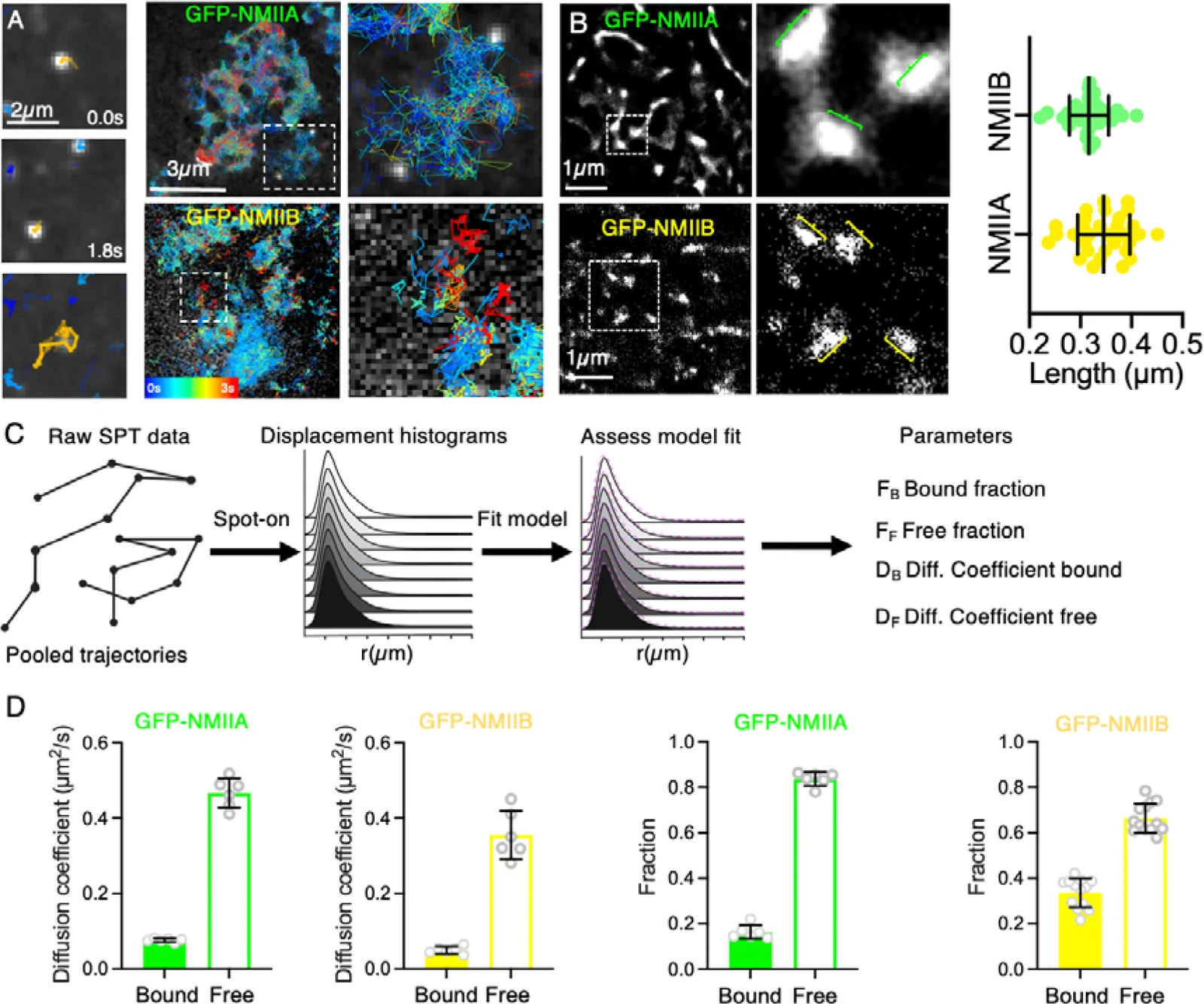
Single-molecule tracking reveals dynamic organization of nonmuscle myosin II at basolateral membrane domains. (A) Left, representative single-particle trajectories of an individual GFP-NMIIA molecule tracked by iSiMM, shown at initial and final time points with local trajectories displayed over adjacent frames. Right, overlay of all GFP-NMIIA and GFP-NMIIB trajectories acquired over 10,000 frames, color-coded by track duration, illustrating spatial confinement of molecular motion within basolateral membrane domains: inset highlights repeated trajectories converging on filament assembly sites. (B) ThunderSTORM super-resolution reconstructions generated from 10,000 frames of iSiMM imaging of GFP-NMIIA and GFP-NMIIB, revealing dense localization clouds corresponding to sites of repeated molecular residency; inset shows representative filament-like localization patterns forming over minutes. Graph shows the length distribution of localization clusters, consistent with reported dimensions of NMII minifilaments. (C) Schematic of the single-molecule analysis workflow illustrating trajectory detection using TrackMate and kinetic modeling using Spot-On. (D) Diffusion coefficients and population fractions of bound and mobile GFP-NMIIA and GFP-NMIIB molecules calculated using a two-state Spot-On model. NMIIA–GFP: N = 3 mice, 6 cells, 391,711 trajectories; GFP-NMIIB: N = 3 mice, 6 cells, 361,614 trajectories.

Single-particle tracking was used to follow the trajectories of individual NMIIA and NMIIB molecules within BLDs (Movie S3). Representative trajectories of a single GFP–NMIIA or GFP–NMIIB molecule are shown in Fig. 2A (left panels), while overlays of multiple trajectories acquired over extended imaging periods reveal spatial confinement of both NMII isoforms within defined regions of the BLM (Fig. 2A, right panels). To assess the spatial organization emerging from these dynamics, super-resolution reconstructions were generated by temporally integrating single-molecule localizations using ThunderSTORM (Ovesný et al., 2014). These reconstructions revealed dense clouds of repeated NMIIA and NMIIB localizations within confined regions, reflecting areas of prolonged molecular residency (Fig. 2B). The longitudinal dimensions of these localization clusters were compatible with the reported size range of NMII minifilaments (Billington et al., 2013), consistent with their identification as sites of NMII assembly.

To quantitatively describe NMII dynamics, single-molecule trajectories were analyzed using a two-state Spot-On model (Hansen et al., 2018) (Fig. 2C). Both isoforms partitioned into mobile and bound populations with distinct diffusion coefficients. NMIIA consisted of a predominant mobile fraction (79.3 ± 5.21%) with a diffusion coefficient of 0.47 ± 0.06 μm^2^/s and a smaller bound fraction (20.7 ± 5.21%) with a diffusion coefficient of 0.076 ± 0.04 μm^2^/s. NMIIB exhibited a larger bound population (33.6 ± 4.19%) and a correspondingly smaller mobile fraction (66.4 ± 4.19%), with diffusion coefficients of 0.37 ± 0.06 μm^2^/s for the mobile fraction and 0.05 ± 0.05 μm^2^/s for the bound fraction (Fig. 2D).

Together, these data indicate that both NMIIA and NMIIB undergo continuous exchange between freely diffusing and localized, low-mobility membrane-proximal states within BLDs. Thus, despite the apparent structural stability of BLDs at the cellular scale, NMII enrichment and higher-order organization are maintained by highly dynamic molecular turnover at the basolateral membrane, a property that is only accessible through single-molecule measurements in vivo.

### NMII-dependent remodeling of basolateral membrane folds during acinar cell expansion

**Figure 3.**
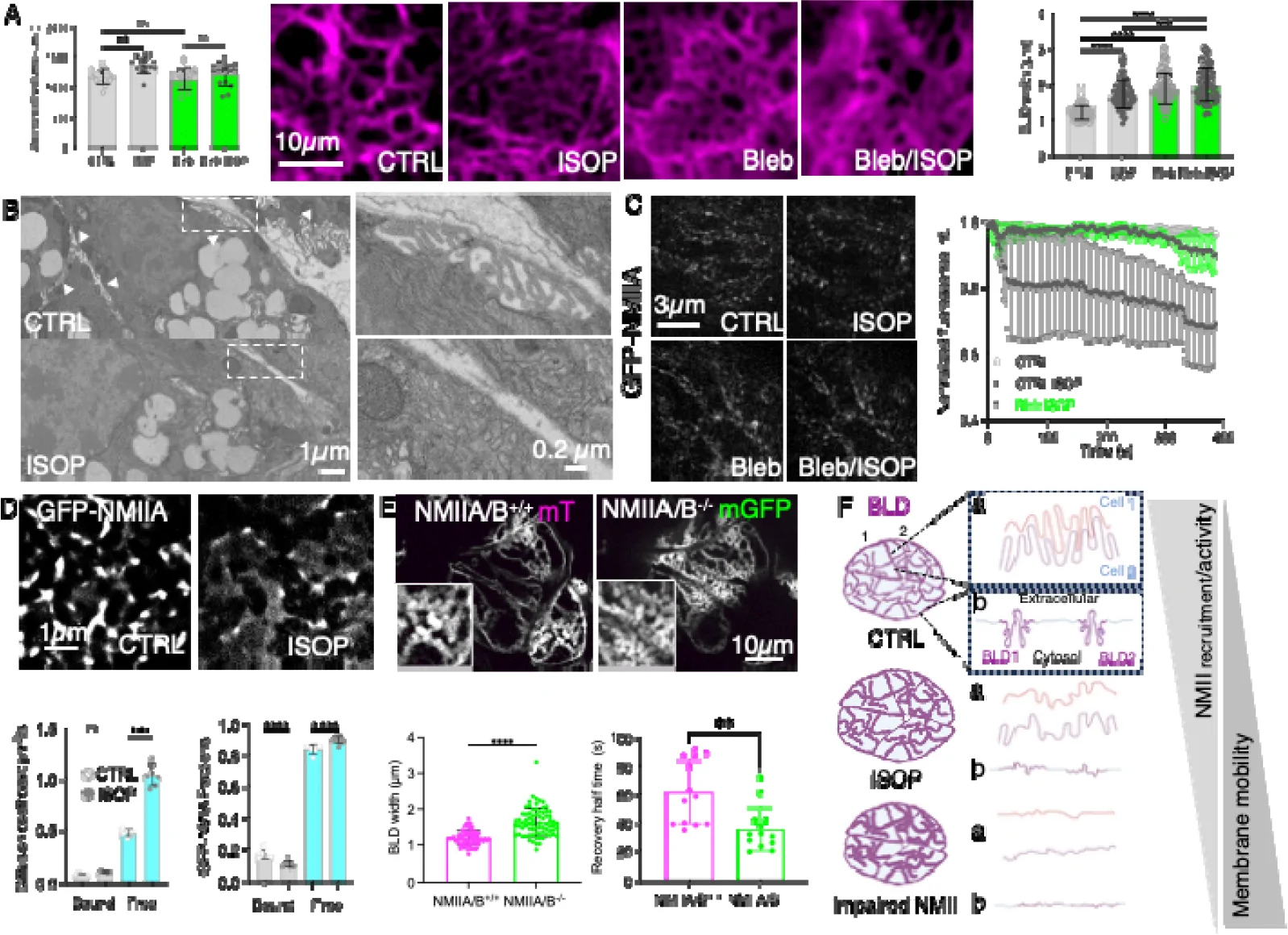
NMII-dependent remodeling of basolateral membrane folds during acinar cell expansion. (A) Left, quantification of acinar cell volume changes before and after β-adrenergic stimulation with isoproterenol (ISOP) in mTom-expressing mice; center, representative single optical sections illustrating changes in basolateral membrane domains (BLD) width under control, ISOP, para-Nitroblebbistatin, and combined ISOP plus para-Nitroblebbistatin conditions, with corresponding quantification of BLD width, indicating release of membrane reserves under stimulation. Scale bar, 10 μm. N= 3 mice, 15 acini per condition. Mean and SD of 15 acini displayed (B) FIB-SEM images of salivary gland acini under control and ISOP-stimulated conditions; arrows indicate deeply folded basolateral membrane architecture in control glands that is reduced following stimulation, with insets highlighting membrane fold organization. (C) Intravital subcellular microscopy (ISMic) of GFP-NMIIA intensity at the basolateral membrane following ISOP stimulation in control (DMSO-treated) and para-Nitroblebbistatin-treated mice, showing a rapid decrease in GFP-NMIIA signal only under control conditions. Mean +/− SD from N= 3 mice, 6 cells per condition are displayed. (D) Intravital single-molecule microscopy (iSiMM) of GFP-NMIIA under control and ISOP-stimulated conditions; ThunderSTORM reconstructions generated from 10,000 frames show reduced spatial persistence of NMIIA localization clusters after stimulation, while Spot-On analysis reveals a marked increase in diffusion of the mobile NMIIA population with minimal change in bound fraction. ISOP NMIIA–GFP: Mean +/− SD from N = 3 mice, 6 cells per condition; 421,238 trajectories total Scale bar, 3 μm. (E) Conditional NMIIA/B-deficient acinar cells identified by membrane GFP expression (NMIIA/B−/−) adjacent to wild-type cells (mTom) show increased BLD thickness compared to controls; FRAP analysis demonstrates faster membrane recovery kinetics in knockout cells, indicating reduced stabilization of membrane-proximal domains in the absence of NMII. Mean +/− SD from N = 3 mice, 12 cells per conditions (F) Schematic model illustrating NMII-dependent stabilization of basolateral membrane folds under resting conditions and turnover-driven membrane unfolding during stimulation, highlighting distinct mechanical responses at cell–cell and cell–extracellular matrix interfaces.

Acinar cells undergo rapid and reversible volume changes during β-adrenergic stimulation (Catalán et al., 2015). Using ISMic imaging of mTom-expressing salivary glands, we quantified acinar volume changes following stimulation with isoproterenol (ISOP). Acini expanded progressively over a 30-minute period, reaching an average increase of ~15% relative to baseline (Fig. 3A, left panel; Fig. S2A).

Inhibition of NMII activity with para-Nitroblebbistatin (Képiró et al., 2014) markedly reduced ISOP-induced acinar expansion (Fig. 3A; Fig. S2A), indicating that NMII activity is required for this physiological response. To assess how inhibition of NMII affects basolateral membrane organization, we quantified the width of basolateral membrane domains (BLDs). Under control conditions, BLDs exhibited an average width of 1.20 ± 0.20 μm. ISOP stimulation increased BLD width to 1.72 ± 0.40 μm, while para-Nitroblebbistatin treatment alone resulted in a width of 1.88 ± 0.43 μm. Combined ISOP and para-Nitroblebbistatin treatment further increased BLD width to 1.99 ± 0.47 μm (Fig. 3A, center images and right panel). These measurements indicate that NMII activity normally constrains BLD architecture under resting conditions and that pharmacological inhibition partially releases this constraint even in the absence of stimulation.

**Figure 4.**
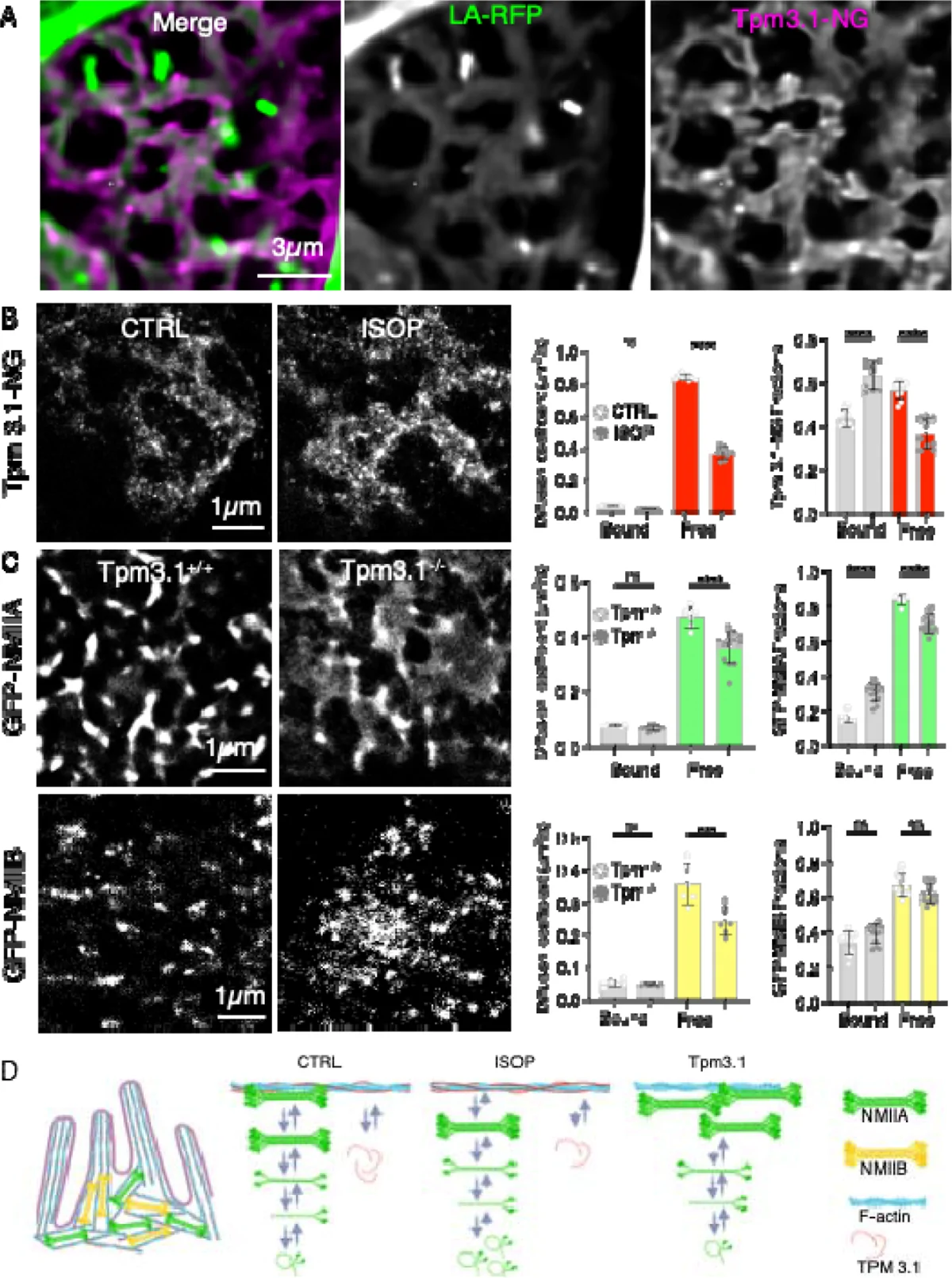
Stimulation-dependent tuning of Tropomyosin 3.1 dynamics regulates NMII molecular exchange and spatial organization. (A) Immunofluorescence staining of salivary glands from NeonGreen–Tpm3.1 knock-in mice (magenta) and F-actin (phalloidin, green) shows enrichment of Tpm3.1 within basolateral membrane domains. Scale bar, 3 μm. (B) ThunderSTORM super-resolution molecular density reconstructions generated from 10,000 frames of intravital single-molecule microscopy (iSiMM) of NeonGreen–Tpm3.1 under control and isoproterenol (ISOP)-stimulated conditions. Tpm3.1 localizes to nanoclusters within basolateral membrane domains that remain spatially confined following stimulation. Spot-On analysis of bound and mobile Tpm3.1 populations shows a significant decrease in diffusion coefficients and a marked increase in bound fraction after ISOP stimulation, indicating enhanced molecular residence without spatial redistribution. Mean +/− SD, from N = 3 mice, 9 cells per condition; 731,941 trajectories total (C) Effects of Tpm3.1 loss on NMII behavior. ThunderSTORM reconstructions generated from 10,000 frames of iSiMM imaging show disorganized spatial enrichment of GFP-NMIIA and GFP-NMIIB in Tpm3.1-deficient acinar cells compared to controls. Spot-On analysis of bound and mobile GFP-NMIIApopulations reveals increased bound fraction and reduced diffusion in the absence of Tpm3.1 (Mean +/− SD, N = 5 mice, 9 cells per condition; 1,235,594 trajectories total). Corresponding analysis of GFP-NMIIB shows increased bound fraction and reduced diffusion of the mobile population in Tpm3.1-deficient cells (Mean +/− SD, N = 5 mice, 9 cells per condition; 925,136 trajectories total). (D) Schematic model illustrating Tpm3.1-dependent kinetic tuning of NMII behavior within basolateral membrane domains. Under stimulation, accelerated molecular exchange limits the persistence of force-generating interactions within preserved domain architecture, promoting dynamic remodeling, whereas loss of Tpm3.1 leads to prolonged residence within low-mobility states, elevated local accumulation, impaired remodeling, and a mechanically rigid, poorly adaptable cortical state.

To examine the ultrastructural organization underlying BLDs, we performed focused ion beam, scanning electron microscopy (FIB-SEM, (Narayan and Subramaniam, 2015). In control glands, the basolateral membrane, both at cell, cell interfaces and at the peripheral edges of acini, was organized into deep membrane folds (Fig. 4B and insets, Movie S4). Following ISOP stimulation, these folded membrane structures were markedly reduced, consistent with unfolding of a pre-existing basolateral membrane reservoir (Fig. 4B, Movie S4). A schematic representation of this organization is shown in Fig. 4F.

We next asked how NMII dynamics change during ISOP stimulation. Monitoring NMIIA, GFP fluorescence intensity at the basolateral membrane revealed a rapid decrease within 5 minutes of ISOP treatment, whereas para-Nitroblebbistatin, treated glands and unstimulated controls showed no change (Fig. 3C). This reduction in fluorescence intensity is consistent with increased NMIIA turnover during stimulation.

Single-molecule measurements supported this interpretation. iSiMM analysis revealed that ISOP selectively increased the diffusion coefficient of the mobile NMIIA fraction, while the bound fraction remained largely unchanged (Fig. 3D, lower panels). ThunderSTORM reconstructions showed that under basal conditions NMIIA formed dense, spatially confined localization clusters, whereas after ISOP stimulation these clusters became more dispersed, with only scattered hotspots of bound NMIIA remaining (Fig. 3D, upper panels).

Together, these data indicate that ISOP accelerates NMIIA binding, unbinding dynamics and promotes partial disassembly of NMII filaments at the basolateral membrane.

The requirement for NMII in maintaining BLD architecture was further examined using conditional NMIIA/B knockout mice (Fig. S2C). In NMIIA/B-deficient acinar cells, BLD thickness was significantly increased compared to wild-type controls, 1.63 ± 0.39 μm versus 1.20 ± 0.20 μm (Fig. 3E, upper panel), indicating exaggerated spreading of basolateral membrane domains in the absence of NMII. Consistent with reduced membrane stabilization, fluorescence recovery after photobleaching (FRAP) analysis revealed faster membrane recovery in NMIIA/B-deficient cells, t½ = 35 ± 8.78 s, compared to wild-type cells, t½ = 49 ± 18.18 s (Fig. 3E, lower right panel).

Together, these results demonstrate that NMII stabilizes folded basolateral membrane architecture under resting conditions, and that NMII-dependent molecular turnover is required for the rapid unfolding of these membrane folds during ISOP-induced acinar cell expansion (Fig. 3F). This framework prompted investigation of upstream factors that regulate NMII kinetics within BLDs.

### Tropomyosin 3.1 exhibits stimulation-dependent kinetic changes and regulates NMII filament organization

To investigate whether tropomyosin isoforms contribute to the regulation of NMII dynamics within basolateral membrane domains (BLDs), we focused on Tropomyosin 3.1 (Tpm3.1), a cytoskeletal regulator known to modulate myosin–actin interactions (Bryce et al., 2003; Gateva et al., 2017; Meiring et al., 2019). Immunofluorescence analysis in a knock-in mouse expressing NeonGreen-tagged Tpm3.1 (Masedunskas et al., 2018), crossed with a LifeAct–RFP F-actin reporter mouse (LF-RFP), revealed strong enrichment of Tpm3.1 within BLDs and close spatial alignment with F-actin (Pearson’s coefficient 0.56 ± 0.05) (Fig. 4A). This confirms that Tpm3.1 localizes to actin-rich basolateral domains in vivo. To quantify Tpm3.1 dynamics at the single-molecule level, we performed intravital single-molecule microscopy (iSiMM) using the NeonGreen–Tpm3.1 knock-in mouse. Under basal conditions, individual Tpm3.1 molecules could be tracked within BLDs, enabling quantitative analysis of their mobility and binding behavior (Fig. 4B). Following ISOP stimulation, Tpm3.1 dynamics were markedly altered. Both the mobile and bound fractions exhibited significantly reduced diffusion coefficients, and the proportion of bound molecules increased by 14.4% relative to basal conditions (Fig. 4B). Despite these kinetic changes, Tpm3.1 remained confined to the same BLDs before and after stimulation, indicating that ISOP strengthens Tpm3.1 engagement with actin filaments rather than redistributing Tpm3.1 within the basolateral membrane.

We next examined how loss of Tpm3.1 affects NMII organization and dynamics. In Tpm3.1-deficient acinar cells, super-resolution reconstructions of NMIIA revealed impaired filament organization, with fewer structured assemblies and a more fragmented distribution compared to controls (Fig. 4C, upper panels). Quantitative single-molecule analysis showed that although the bound fraction of NMIIA increased in the absence of Tpm3.1, the mobility of the free NMIIA population was reduced (Fig. 4C, lower panels), indicating altered NMIIA exchange dynamics.

NMIIB exhibited a similar phenotype in Tpm3.1-deficient cells. Loss of Tpm3.1 resulted in reduced diffusion of the mobile NMIIB fraction and pronounced disruption of filament organization, as revealed by super-resolution reconstructions (Fig. 4C, upper and lower panels). Together, these data indicate that Tpm3.1 does not simply recruit NMII to actin but instead modulates both the dynamic properties and the structural organization of NMII filaments within BLDs.

## Discussion

A longstanding limitation in cell biology has been the inability to directly measure molecular kinetics within intact mammalian tissues, forcing mechanistic inference to rely largely on cultured cells or reconstituted systems where geometry, mechanical constraints, and physiological context differ substantially from those in vivo. By establishing intravital single-molecule microscopy (iSiMM), we directly quantify molecular binding and diffusion of endogenous cytoskeletal proteins in a living mouse. To our knowledge, this represents the first demonstration of single-molecule tracking of cytoskeletal components in vivo, enabling kinetic measurements previously inaccessible in mammalian tissues.

Using intravital subcellular microscopy (ISMic), we identify discrete basolateral membrane domains built on deeply folded membrane infolds in salivary gland acinar cells. These domains are morphologically stable yet maintained by rapid molecular exchange of NMIIA, NMIIB, and Tpm3.1, and ultrastructural analysis confirms that they constitute a pre-existing membrane reservoir rather than sites of basolateral exocytosis (Cosen-Binker et al., 2008). Accordingly, acinar cell expansion during β-adrenergic stimulation occurs primarily through deployment of stored membrane rather than membrane addition, consistent with the apical restriction of secretion-associated exocytosis (Masedunskas et al., 2011a).

Single-molecule measurements obtained by iSiMM reveal that membrane deployment is governed by kinetic regulation rather than large-scale architectural reorganization. β-adrenergic stimulation preserves the spatial arrangement of basolateral membrane domains but leads to a partial (≈20–30%) reduction in NMIIA occupancy at the micron scale. At the molecular level, this reduction is accompanied by increased diffusion of the free NMIIA population and reduced spatial persistence of NMII enrichment zones, indicating that individual NMII molecules exit localized, low-mobility states near the basolateral membrane more rapidly. Although higher-order assemblies may still form transiently within the existing architecture, shortened molecular residence is expected to limit the persistence of force-generating interactions. Together, these observations identify accelerated molecular exchange and reduced occupancy, rather than domain relocation or dissolution, as the principal determinants of membrane fold unfolding during stimulation.

These findings highlight that persistent NMII residence and rapid NMII turnover have opposing mechanical consequences. Under resting conditions, sustained residence of NMII within localized membrane-proximal states stabilizes folded membrane architecture by maintaining long-lived contractile constraints, consistent with established models of actomyosin-based cortical tension (Wang et al., 2023). In contrast, accelerated molecular exchange combined with reduced local occupancy shortens the lifetime of force-bearing interactions, promoting stress relaxation and fluidization of the basolateral cortex, a transition characteristic of active actomyosin networks with high motor turnover (Chugh et al., 2017; Murrell and Gardel, 2012; Salbreux et al., 2012). Thus, membrane remodeling is controlled by the timescale and persistence of NMII engagement rather than by absolute NMII abundance or equilibrium occupancy alone.

Tropomyosin 3.1 emerges as a critical regulator of this kinetic balance. Upon stimulation, Tpm3.1 becomes more stably associated with F-actin, as reflected by increased bound fraction and reduced diffusion, without altering its spatial distribution. This stabilization correlates with accelerated NMII molecular exchange, indicating that Tpm3.1 modulates the properties of membrane-proximal cytoskeletal states to permit rapid, productive turnover, consistent with prior work showing that tropomyosin isoforms tune myosin II kinetics and mechanical output (Gateva et al., 2017; Meiring et al., 2019; Wang et al., 2023). In contrast, loss of Tpm3.1 shifts NMII into prolonged residence within localized, low-mobility states, leading to increased binding, reduced diffusion, and disorganized spatial enrichment. Despite elevated local accumulation, reduced molecular exchange limits effective remodeling, resulting in a cortex that is mechanically rigid and poorly adaptable rather than dynamically responsive (Alvarado et al., 2013; Banerjee et al., 2017).

Together, these findings support a model in which Tpm3.1 acts as a kinetic gatekeeper, tuning NMII residence times and occupancy to regulate transitions between force-maintaining and remodeling states. Persistent NMII engagement stabilizes membrane folds, whereas accelerated molecular exchange coupled with reduced occupancy promotes stress relaxation and membrane deployment. Although we cannot formally exclude contributions from membrane trafficking to membrane homeostasis, intravital subcellular microscopy detects no basolateral exocytic events under the conditions examined, and ultrastructural analysis supports deployment of a pre-existing membrane reservoir through unfolding rather than membrane addition, suggesting that trafficking-independent mechanisms dominate the acute response.

While the current implementation of iSiMM is limited by imaging depth, this constraint reflects optical access rather than conceptual scope. In practice, iSiMM is well suited for interrogating molecular kinetics in tissues where the biology of interest lies near the surface of intact organs, and future advances in intravital optics are expected to extend this reach. More broadly, iSiMM provides a general framework for linking molecular kinetics to tissue-level physiology in living animals, extending single-molecule biophysics into its native physiological context.

## Materials and Methods

### Animal strains and procedures

All animal experiments were conducted in accordance with guidelines from the National Cancer Institute (NIH, Bethesda, MD, USA) and the NSW Animal Research Act (1985) and approved by the respective institutional animal care and ethics committees. Male and female mice (8–24 weeks old) were maintained on standard chow and water ad libitum.

The mT/mG Cre reporter mouse was obtained from Jackson Laboratory. Hemizygous LifeAct-GFP and LifeAct-RFP mice were provided by R. Wedlich-Soldner (Riedl et al., 2010). GFP–NMIIA knock-in mice were generated as described (Zhang et al., 2012) and crossed with LifeAct-RFP mice. The endogenous Tpm3.1–mNeonGreen knock-in mouse line [B6-Tpm3tm5(mNeonGreen)Hrd] was generated as described (Masedunskas et al., 2018). Conditional NMIIA/B floxed mice (NMIIA/Bfl/fl) (Deguchi et al., 2016) were crossed with Mist1CreERT2 mice (Aure et al., 2015) and the mT/mG reporter strain. Cre recombination was induced by two intraperitoneal injections of tamoxifen (75 mg/kg body weight) administered two days apart. Mice were imaged 3–8 weeks after induction. Recombined (GFP-positive) and non-recombined (mTom-positive) acinar cells were distinguished by fluorescence.

### Intravital subcellular microscopy (ISMic)

Mice (18–34 g) were anesthetized by intraperitoneal injection of ketamine (100 mg/kg) and xylazine (15 mg/kg). Submandibular salivary glands were surgically exteriorized and stabilized on a coverslip mounted on the microscope stage as previously described (Masedunskas et al., 2011). Glands were maintained with intact vasculature and innervation during imaging.

### β-adrenergic stimulation and inhibitor treatment

Secretory stimulation was induced by subcutaneous injection of isoproterenol (ISOP; 0.01 mg/kg). For inhibition experiments, exposed glands were bathed in 50 μM para-Nitroblebbistatin (in 1.5% DMSO saline) for 10 min before stimulation. Control glands received vehicle alone. For perfusion experiments, solutions were applied at 10 μL/min using a programmable syringe pump.

### Intravital single-molecule microscopy (iSiMM)

Single-molecule imaging was performed on a Nikon Ti microscope equipped with a custom highly inclined and laminated optical sheet (HILO) illumination path. Excitation was provided by a 488-nm laser expanded and focused onto the back focal plane of the objective. The incidence angle (80–85°) was optimized empirically to maximize signal-to-noise at imaging depths of ~15–30 μm. Images were acquired at 30 ms per frame.

### iSIM imaging

iSIM imaging was conducted on Nikon Ti body base integrated with VT-iSIM (VisiTech International) module. Z-stacks of samples were acquired using a 100x oil immersion objective lens (Nikon NA 1.49) and sCMOS camera (ORCA-Quest; Hamamatsu). Lasers, cameras, stages, triggering, and microscopy are all controlled through Nikon NIS-Elements Imaging software. 488-nm laser was employed to excite GFP with a 525/50 bandpass filter.

### Spinning disk confocal microscopy

High-resolution 3D imaging was performed on a Nikon Ti2 spinning disk microscope. Z-stacks were acquired at 0.05–0.3 μm intervals with exposure times of 80–300 ms. Image alignment, denoising, and 3D deconvolution (Richardson–Lucy algorithm) were performed using Nikon Elements software.

### Image processing and analysis

Image processing was performed using Fiji/ImageJ, Nikon Elements, Imaris, and PRISM. Drift and motion was minimized by stabilizing the external gland as described before (Ebrahim et al., 2019; Heydecker et al., 2024; Masedunskas et al., 2011a). When required, drift correction was performed using StackReg. Where required, Nikon Denoise.ai and Richardson–Lucy deconvolution were applied.

### Single-molecule detection and tracking

Emitter detection and trajectory reconstruction were performed using TrackMate (Ershov et al., 2022) with a Laplacian of Gaussian detector (estimated diameter 0.6 μm), sub-pixel localization, and median filtering. Tracks were generated using a simple LAP tracker (maximum linking distance 0.9 μm; maximum frame gap 2). Tracks shorter than four frames were excluded. Trajectories were exported to Spot-On (Hansen et al., 2018) for two-state kinetic modeling (bound/free model, localization error fitted from data).

### ThunderSTORM reconstruction and filament measurements

Super-resolution density reconstructions were generated using ThunderSTORM (Ovesný et al., 2014) from 10,000-frame datasets with default parameters and cross correlative drift correction. Filament lengths were measured in Fiji along line profiles until signal intensity returned to baseline.

### Volumetric measurements

Acinar volumes were segmented manually using Imaris (v9.2.1) surface reconstruction tools based on membrane fluorescence. Surface volumes were exported and analyzed in PRISM.

### Basolateral membrane domain (BLD) width measurements

BLD width was measured using perpendicular 2s in Fiji. Width was defined as the distance between intensity plateaus at which mTom signal sharply decreased.

### NMII intensity measurements

Z-stacks (3 μm) were acquired to compensate for drift. Maximum projections were generated, and fluorescence intensity at BLDs was measured over time in Fiji. Data were normalized and analyzed in PRISM.

### FRAP analysis

FRAP experiments were performed using a 405-nm bleaching laser on the spinning disk system. Recovery was recorded for 8 min as 3-μm Z-stacks. Intensity was measured in Fiji and analyzed using easyFRAP to determine half-times of recovery.

### Immunofluorescence

Cardiac perfusion was performed with PBS followed by 4% formaldehyde. Glands were cryoprotected in sucrose, embedded in OCT, and sectioned (10 μm). Sections were permeabilized (0.5% Triton X-100), blocked (10% NGS), and incubated with primary antibodies overnight at 4°C, followed by secondary antibodies. Slides were mounted in Fluormount-G.

### Tissue sample preparation and FIB-SEM

Mice were anesthetized and for stimulated group treated with ISOP. Salivary glands were surgically exposed, excised, washed in saline and fixed with Karnovsky’s fixative for 2 hours and then post-fixed in 2% osmium tetroxide with 1.5% potassium ferricyanide in 0.1 M sodium cacodylate buffer for 1 h at room temperature. Tissues were washed, stained overnight with 1% aqueous uranyl acetate at 4°C, washed again, and treated with lead aspartate at 65°C for 30 min. Tissues were dehydrated in graded ethanol (35%, 50%, 70%, 95%, and 100% x 5; 10 min each) and propylene oxide (PO; 100% × 5; 10 min each), infiltrated with Polybed 812 resin (resin: PO; 1:3, 1:2, 3:1, and 100%), embedded, and polymerized at 65°C for 48 h. Resin blocks were mounted on pin stubs, silver painted, and carbon coated (10 nm) before imaging in a Zeiss Crossbeam 550 FIB-SEM. Regions of interest were identified using a 1.5 kV, 1.1 nA SEM beam. FIB preparation followed the ATLAS3D workflow (Fibics, Ottawa, Canada), including a 1 μm platinum pad deposition, milling of tracking and autofocus lines before carbon coating, and trench milling with a 30 nA beam followed by 1.5 nA polishing. SEM images were acquired at 1.5 kV, 1.1 nA with 4 nm pixel size and 8 nm slice thickness using an in-lens EsB detector (grid 790 V). Image stacks were registered, inverted, and cropped in Atlas 5, then binned in 3dmod to generate 3D volumes at 8 nm isotropic resolution.

### Statistical analysis

A minimum of three independent animals and six cells per condition were analyzed unless otherwise indicated in the figure legends. For all analyses, biological replicates were defined at the animal level. Measurements obtained from individual cells were averaged per animal prior to statistical comparison. Comparisons were performed between animal means unless otherwise specified.

Statistical analyses were conducted using Prism 10 (GraphPad Software). For comparisons between two conditions, unpaired two-tailed Student’s *t*-tests were performed on animal means. For comparisons involving more than two conditions, one-way ANOVA was performed on animal means, followed by appropriate post hoc testing where applicable.

Data are presented as mean ± SD unless otherwise stated. Statistical significance was defined as: ns, *P* > 0.05; *, *P* ≤ 0.05; **, *P* ≤ 0.01; ***, *P* ≤ 0.001; ****, *P* ≤ 0.0001.

## Supplementary Material

Supplement 1

Supplement 2

Supplement 3

Supplement 4

Supplement 5

Supplement 6
