# Supplementary material for: Intravital single-molecule imaging reveals cytoskeletal turnover as a driver of membrane remodeling in live animals
Source: bioRxiv. 2026 Feb 25:2026.02.25.708035. Preprint. [Version 1] doi: 10.64898/2026.02.25.708035 (PMC12970338; doi:10.64898/2026.02.25.708035)
Supplement: Supplementary file 1 [file NIHPP2026.02.25.708035v1-supplement-6.pdf]

bioRxiv preprint doi: https://doi.org/10.64898/2026.02.25.708035; this version posted February 25, 2026. The copyright holder for this preprint (which was not certified by peer review) is the author/funder. This article is a US Government work. It is not subject to copyright under 17 USC 105 and is also made available for use under a CC0 license.

Supplementary Figures

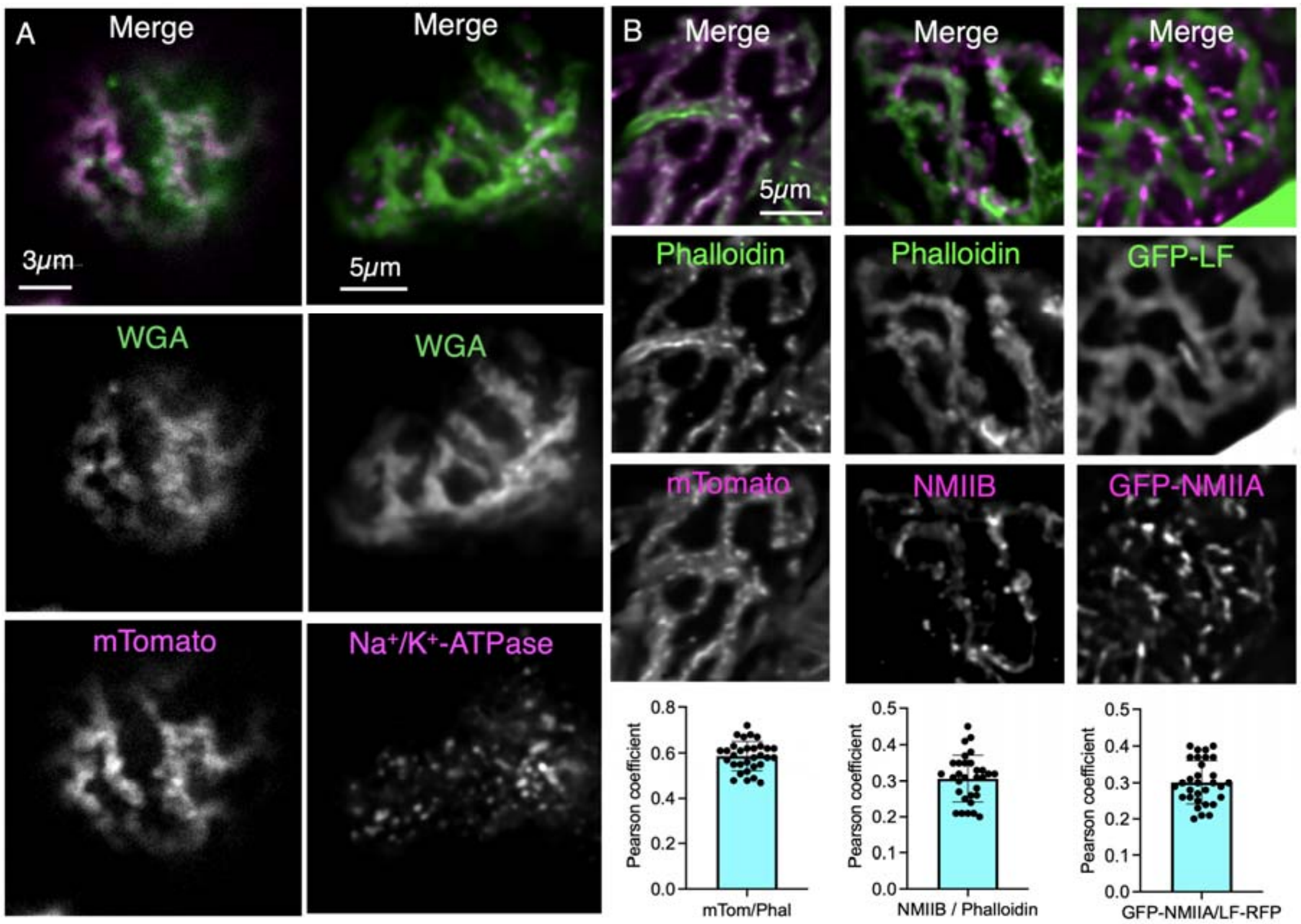

Figure S1. Basolateral membrane domains colocalize with membrane and cytoskeletal markers. (A) Immunofluorescence analysis of salivary glands from mTom mice shows colocalization of the membrane-targeted tdTomato reporter (magenta) with wheat germ agglutinin (WGA, green), confirming enrichment of membrane signal within basolateral membrane domains. In wild-type mice lacking membrane reporters, WGA labeling colocalizes with Na^+/K^+-ATPase, a canonical basolateral membrane marker, confirming the basolateral identity of these domains. Scale bars, 3 and 5 µm. (B) Left, mTom-expressing glands counterstained with phalloidin demonstrate enrichment of F-actin within basolateral membrane domains, with Pearson's correlation coefficients calculated to quantify colocalization. Middle, wild-type glands stained for NMIIB (magenta) and F-actin confirm association of NMII filaments with basolateral membrane domains. Right, GFP-NMIIAcrossed with LifeAct–RFP mice show spatial alignment of NMIIA with actin-rich membrane domains, with Pearson's coefficients indicating significant colocalization, For each conditions Means +/- SD, from N = 3 mice, 25 cells are shown.

bioRxiv preprint doi: https://doi.org/10.64898/2026.02.25.708035; this version posted February 25, 2026. The copyright holder for this preprint (which was not certified by peer review) is the author/funder. This article is a US Government work. It is not subject to copyright under 17 USC 105 and is also made available for use under a CC0 license.

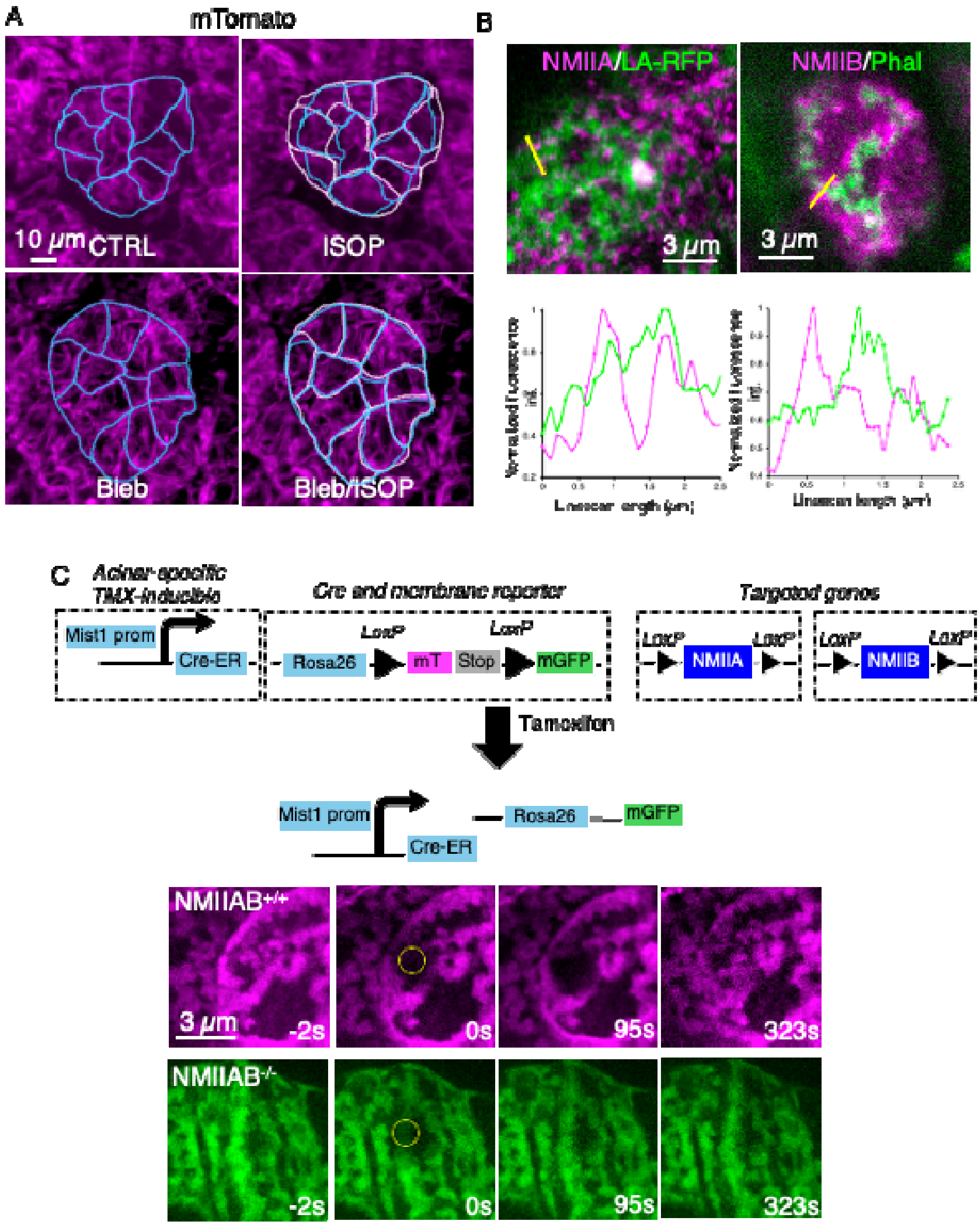

Figure S2. NMII activity contributes to basolateral membrane organization and acinar cell expansion. (A) Representative intravital images of acinar cell from mTom-expressing mice before (light blue outline) and after β-adrenergic stimulation with isoproterenol (ISOP; pink outline), showing ~15% volume expansion. Treatment with para-Nitroblebbistatin inhibits cell expansion while increasing basolateral membrane domain width, consistent with altered membrane deployment. Scale bar, 10 μm. (B) Line scans across basolateral membrane domains (BLDs) in NMIIA/LifeAct-RFP mice and in NMIIB/phalloidin-stained sections. Yellow bars indicate the position of the line scan. NMII signal is enriched at the periphery of BLDs, peaking adjacent to, but preceding, the peak actin signal, which is strongly associated with the domains. (C)

Schematic of the conditional NMIIA/B knockout strategy. Mice expressing Cre recombinase under the acinar-specific Mist1 TMX-inducible promoter were crossed with NMIIA/B floxed alleles and the mTom/mGFP membrane reporter. Following tamoxifen treatment, acinar cells expressing Cre are GFP-positive and lack NMIIA/B. Intravital subcellular microscopy and fluorescence recovery after photobleaching (FRAP) analysis were performed on adjacent NMIIA/B-deficient (mGFP) and control (mTom) cells to assess the contribution of NMII to membrane-proximal domain organization and recovery kinetics.
