# Supplementary material for: Intravital single-molecule imaging reveals cytoskeletal turnover as a driver of membrane remodeling in live animals
Source: bioRxiv. 2026 Feb 25:2026.02.25.708035. Preprint. [Version 1] doi: 10.64898/2026.02.25.708035 (PMC12970338; doi:10.64898/2026.02.25.708035)
Supplement: Supplementary file 2 [file media-1.docx]

**Legends to Supplementary Movies**

**Movie S1**

Six-minute time-lapse imaging of a 2.5-µm Z-stack of the basolateral membrane of an acinus in a mouse expressing mTom (magenta). Z-stacks were acquired by spinning-disk confocal microscopy.

**Movie S2**

Single optical planes extracted from a 2.5-µm Z-stack of the basolateral membrane of an acinus in a mouse expressing mTom (magenta). Basolateral membrane domains (BLDs) are visible as described in Fig. 1A. Z-stacks were acquired by spinning-disk confocal microscopy.

**Movie S3.**

Intravital single-molecule microscopy (iSiMM) time-lapse imaging of basolateral membrane domains (BLDs) in NMIIA–GFP and NMIIB–GFP mice. The left panel shows NMIIA–GFP molecules tracked by iSiMM; the right panel shows NMIIB–GFP molecules. Bottom panels display local molecular trajectories overlaid using TrackMate and color-coded by track duration, illustrating spatial confinement of molecular motion adjacent to BLDs. Experiments were performed in 3 NMIIA–GFP mice and 5 NMIIB–GFP mice.

**Movie S4.**

isoproterenol (ISOP)-stimulated mice. Following ISOP stimulation, the folded membrane architecture underlying BLDs is markedly reduced, as described in Fig. 3B. Experiments were performed in 2 control mice and 2 ISOP-stimulated mice.
